# Analysis of the threshold image contrast obtained with the CDMAM 3.4 and CDMAM 4.0 phantoms

**DOI:** 10.1007/s13246-023-01264-1

**Published:** 2023-04-25

**Authors:** Michał Biegała, Teresa Jakubowska, Anna Stępińska, Piotr Woźniak

**Affiliations:** 1 Faculty of Medicine, Department of Medical Imaging Technology, Medical University of Lodz, Lindleya 6, Lodz, 90-131 Poland; 2 Department of Medical Physics, Copernicus Memorial Hospital in Lodz Comprehensive Cancer Center and Traumatology, Lodz, Poland; 3 Institute of Applied Radiation Chemistry, Lodz University of Technology, Lodz, Poland

**Keywords:** Quality control, Mammography, Threshold image contrast, CDMAM phantom

## Abstract

Quality control in mammography is a very important element. One of the parameters indicating the appropriate image quality is the threshold image contrast. The CDMAM phantom is used to measure this parameter. It is currently available in two versions 3.4 and 4.0. The aim of this work is to compare the threshold image contrast readings obtained with the CDMAM 3.4 and CDMAM 4.0 phantoms. In the measurements, 9 CDMAM 4.0 phantoms were used to check the difference in indications of individual copies. The phantom whose readings were closest to the average of all readings was used for comparative measurements with the CDMAM 3.4 phantom. Measurements were made on 40 mammography devices. The obtained images were read with the software provided by the phantom manufacturer and the CDMAM Analysis v2.3.0 (NCCPM) software. The average percentage difference between the minimum and maximum values indicated by the CDMAM 4.0 phantoms was 10.09%. Using the CDMAM Analysis v2.3.0 (NCCPM) software, the average difference in readings between the CDMAM 3.4 and CDMAM 4.0 phantoms is 7.93%, and when using the software provided by the phantom manufacturer, it is as much as 60.15%. The obtained results of the threshold image contrast are affected by the type of software used for reading and the accuracy of the execution of individual elements of the phantom. It is recommended to use CDMAM Analysis v2.3.0 (NCCPM) software or the latest software provided by the phantom manufacturer to read the phantom images.

## Introduction

Quality control in mammography, and especially in screening mammography, is an essential element to ensure high quality of the images obtained and a sufficiently low dose that the examined patients receive [1]. European guidelines and national legal regulations define the scope and frequency of quality control tests in the field of digital mammography [2]. Image quality and dose are key parameters to obtain a reliable result with a high level of patient safety. Appropriate measuring equipment and specialized phantoms are needed to assess key quality parameters in digital mammography [3, 4]. A particularly difficult parameter to measure is the threshold image contrast, which allows to assess the quality of the digital image obtained during mammography [5]. The analysis of this parameter requires the use of appropriate phantoms that allow the measurement of the threshold image contrast in an objective and independent of the human eye [6]. One such phantom is the CDMAM phantom. The CDMAM phantom from Artinis is available in two versions: CDMAM 3.4 and CDMAM 4.0. In this study, the consistency of the results obtained from different CDMAM 4.0 phantoms was measured and the readings of CDMAM 3.4 and CDMAM 4.0 phantoms on different mammography machines were compared. Phantom readings were taken using software provided by the phantom manufacturer and another commercially available. The size of ionizing radiation doses emitted by mammography machines during tests with the use of CDMAM phantoms was also analyzed, depending on the results obtained. This allows us to characterize the functioning of individual mammography machines in terms of the emitted doses of ionizing radiation, which is particularly important in the process of optimizing the radiological protection of the examined women.

## Materials and methods

CDMAM phantoms are used to measure the threshold image contrast, which is defined as a function of the object diameter, by detecting pairs of low-contrast objects [7, 8]. In the CDMAM 3.4 phantom, the objects (gold discs) have a diameter of 0.10 to 2.30 mm and a thickness of 0.05 to 1.60 μm, which in the conditions of anode/filter Mo/Mo gives a contrast range of 1–25% [9]. The continuous improvement of image quality in mammography (e.g. other anode/filter) has made it necessary to visualize less contrast as well as higher spatial resolution (smaller objects). The CDMAM 4.0 version of the phantom has been modified [10]. The gold discs used in this system have a diameter of 0.06 to 2.00 mm and a thickness of 0.03 to 2.00 μm, which gives a contrast range of 0.5–30% in standard mammography exposure conditions [11]. In the case of CDMAM 4.0, the contrast curve of the assessed mammography system is determined with greater accuracy [12]. The CDMAM 3.4 phantom contains 205 cells and the CDMAM 4.0 phantom contains 336 cells placed on an aluminum plate (99.5% aluminum), with one middle and one corner gold disk of a certain diameter and thickness in each cell. The corner where the disk is located changes randomly between cells. The combination of disc diameter and thickness is unique to each cell. Each phantom has 4 PMMA plates with a thickness of 10.0 ± 0.1 mm each to maintain dispersion conditions. The total thickness of the phantom corresponds to 6 cm of the breast after compression. The phantoms differ not only in the number of cells with golden discs, but also in their geometric arrangement on the tile. Each of the phantoms allows you to determine the threshold contrast of discs with a diameter of 0.10 mm, 0.25 mm, 0.50 mm, 1 mm, 2 mm. Discs of the same diameter allow contrast values to be compared using the CDMAM 3.4 and CDMAM 4.0 phantoms [13–16, 17], as recommended by the European Reference Organization for Quality Assured Breast Screening and Diagnostic Services. 9 phantoms were used to analyze the comparison of CDMAM 4.0 phantom readings. The comparison was made on a Selenia Dimensions mammography machine by Hologic with a digital detector based on a-Se (DR). The parameters used for the exposure of the phantoms were W/Ag, 31 kV, 120 mAs (the parameters correspond to the clinical exposure of 5 cm PMMA with a 1 cm spacer). For each phantom, 16 images were taken (for processing). The CDMAM4 Analyzer 2.4.0.1 software from Artinis was used to read the obtained images. Comparative analysis of image contrast threshold readings using CDMAM 3.4 and CDMAM 4.0 phantoms was performed on 40 DR and CR mammography machines:

- 20 Siemens Mammomat Fusion mammographs with a-Si (DR) based detector,
- 12 Planmed Clarity S with a-Si (DR) based detector,
- 4 Planmed Sophia Classic with a high-resolution phosphor plate detector (CR),
- 1 FujiFilm’s Amulet Innovality with a-Se (DR) based detector,
- 2 Hologic Selenia with a-Se (DR) based detector and.
- 1 Hologic Selenia Dimensions with a-Se (DR) based detector.

The CDMAM Analysis v2.3.0 software from the National Coordinating Center for the Physics of Mammography, Guildford, UK (NCCPM) was used to read the obtained images [18]. The results were also compared using the software supplied with the phantoms by the manufacturer Artinis, i.e., CDMAM Analyser 1.2 and CDMAM4 Analyser 2.4.0.1, respectively. Due to the format of saved images, the comparative analysis with the original software was performed on 23 DR mammography machines:

- 20 Siemens Mammomat Fusion mammographs with a-Si (DR) based detector,
- 1 Amulet Innovality by Fuji Film with an a-Se (DR) based detector,
- 2 Hologic Selenia with an a-Se (DR) based detector.

The parameters used for the exposure of the phantoms correspond to the clinical exposure of 5 cm PMMA with a 1 cm spacer. For the comparative analysis, a phantom was used whose threshold image contrast results were closest to the average obtained from the reading analysis of 9 CDMAM 4.0 phantoms.

The operation of the CDMAM Analyser 1.2 software is based on the CDCOM 1.5.2 source file, while the operation of the CDMAM4 Analyser 2.4.0.1 software is based on the CDCOM 4.1.0 file. The CDMAM Analysis v2.3.0 software uses both CDCOM 1.6 and CDCOM 4.1.0 files for its calculations, since the software is capable of analyzing both versions of CDMAM phantoms. It should be noted that software from different manufacturers may be based on the same CDCOM source file (detecting gold discs in the image), however, the data obtained from this file may be analyzed in different ways by individual software, which affects the result of the analysis.

Many mammography machines used in this work allows to analyze the dependence of the readings obtained with the tested CDMAM phantoms depending on the dose of ionizing radiation falling on the detector. Values obtained from CDMAM phantoms for gold discs with a diameter of 0.10 mm were analyzed. Siemens and Planmed mammographs were analyzed due to their large number used in this study. CDMAM Analysis v2.3.0 (NCCPM) software was used in this analysis.

Statistical analysis of the results obtained in this work was carried out using the Statistica 13.1 software. The statistical tool used to compare the two groups of results is the Student’s t-test for dependent samples. In this test, the level of significance was α = 0.05, so P < 0.05 confirms the statistical difference between the tested samples.

## Results

Table 1 presents the results of the analysis of readings from 9 CDMAM 4.0 phantoms. The table shows the average readings of the threshold image contrast obtained from 9 CDMAM 4.0 phantoms for each diameter of the gold discs along with the doubled standard error of the reading, the minimum and maximum values of the obtained readings, and the difference between the minimum and maximum values for individual readings, expressed as a percentage.

**Table 1:** Analysis of threshold image contrast obtained from 9 CDMAM 4.0 phantoms

diameter gold disc [mm]	average value – threshold image contrast ± 2*SE	maximum value - threshold contrast	minimum value - threshold contrast	difference (max-min)/min [%]
0.08	1.111 ± 0.075	1.181	1.059	11.60
0.09	0.906 ± 0.059	0.963	0.864	11.38
0.10	0.758 ± 0.052	0.804	0.725	10.87
0.13	0.493 ± 0.029	0.521	0.470	10.77
0.15	0.396 ± 0.023	0.414	0.375	10.48
0.18	0.303 ± 0.017	0.314	0.286	9.80
0.21	0.244 ± 0.014	0.255	0.231	10.52
0.25	0.194 ± 0.011	0.204	0.184	10.80
0.30	0.155 ± 0.009	0.163	0.147	10.60
0.35	0.129 ± 0.007	0.136	0.124	10.12
0.42	0.106 ± 0.006	0.111	0.102	9.27
0.50	0.088 ± 0.005	0.093	0.084	9.93
0.57	0.078 ± 0.004	0.081	0.074	10.31
0.66	0.068 ± 0.004	0.071	0.064	10.45
0.77	0.060 ± 0.003	0.062	0.056	10.28
0.88	0.053 ± 0.003	0.055	0.050	9.88
1.00	0.049 ± 0.002	0.050	0.046	9.31
1.20	0.043 ± 0.002	0.044	0.041	8.22
1.40	0.039 ± 0.002	0.040	0.037	7.61
1.70	0.035 ± 0.002	0.036	0.033	8.87
2.00	0.032 ± 0.002	0.033	0.030	10.81

Table 2 presents the average values of the threshold image contrast with double standard error obtained for two versions of CDMAM phantoms on 40 mammography machines. Threshold image contrast were obtained using CDMAM Analysis v2.3.0 (NCCPM) software.

**Table 2:** Comparison of threshold image contrast for CDMAM 3.4 and CDMAM 4.0 phantoms using CDMAM Analysis v2.3.0 (NCCPM) software

diameter gold disc [mm]	phantom CDMAM 3.4 average value – threshold image contrast ± 2*SE	phantom CDMAM 4.0 average value – threshold image contrast ± 2*SE
0.10	1.096 ± 0.053	1.062 ± 0.049
0.25	0.250 ± 0.008	0.272 ± 0.012
0.50	0.111 ± 0.004	0.120 ± 0.005
1.00	0.057 ± 0.002	0.064 ± 0.003

Table 3 presents the average values of the threshold image contrast with double standard error obtained for two versions of CDMAM phantoms on 23 mammography machines. Threshold image contrast were obtained using CDMAM Analyser 1.2 and CDMAM4 Analyser 2.4.0.1 – Artinis software.

**Table 3:** Comparison of threshold image contrast for CDMAM 3.4 and CDMAM 4.0 phantoms using CDMAM Analyser 1.2 and CDMAM4 Analyser 2.4.0.1 – Artinis software

diameter gold disc [mm]	phantom CDMAM 3.4 average value – threshold image contrast ± 2*SE	phantom CDMAM 4.0 average value – threshold image contrast ± 2*SE
0.10	0.911 ± 0.051	1.100 ± 0.040
0.25	0.175 ± 0.011	0.271 ± 0.008
0.50	0.068 ± 0.003	0.122 ± 0.004
1.00	0.035 ± 0.002	0.065 ± 0.002

## Discussion

At the outset, it is worth noting that the available literature did not compare the readings of as many as 9 phantoms of the same type and did not compare the readings of the CDMAM 3.4 and CDMAM 4.0 phantoms on 40 mammography machines from as many as 6 different manufacturers equipped with different image detectors.

The analysis of the obtained readings from 9 CDMAM 4.0 phantoms showed an average difference between phantoms of 10.09%. Analyzing Table 1, the differences between the phantoms change for each thickness of the gold disc. The greatest difference in the reading was observed for the 0.08 mm diameter disc and it was 11.60%, and the smallest for the 1.4 mm disc was 7.61%. The average difference in the values obtained for discs with a diameter of 0.10 mm, 0.25 mm, 0.50 and 1.00 mm, which have their reference values according to the European protocol, is 10.23%. In the article by Fabiszewska et al. [19], the readings from 3 CDMAM 3.4 phantoms were analyzed on two mammography machines. This article had an average percentage difference between the minimum and maximum values of the CDMAM 3.4 phantoms of 12%. In the case of the CDMAM 4.0 phantom, the differences in readings for individual copies are smaller. This may indicate an improvement in the method of manufacturing individual copies of the phantom and improvement of the software for reading CDMAM 4.0 phantom images.

Comparative analysis of the threshold image contrast obtained with the CDMAM 3.4 and CDMAM 4.0 phantoms was performed for four diameters of gold discs (Table 2). Using CDMAM Analysis v2.3.0 (NCCPM) software for a gold disc diameter of 0.10 mm, the difference in reading between CDMAM 3.4 and CDMAM 4.0 was 3.2% (p = 0.359), for gold disc diameters of 0.25 mm, 0.50 and 1.00 mm, these differences were 8.8% (p = 0.003), 8.3% (p = 0.003) and 11.4% (p = 0.000), respectively. It is worth noting that for a disk diameter of 0.1 mm, the threshold image contrast value obtained with the CDMAM 3.4 phantom was 3.2% higher than the value obtained with the CDMAM 4.0 phantom.

For the remaining diameters of gold discs, the trend was reversed, i.e., the values obtained with the CDMAM 3.4 phantom were lower than those obtained with the CDMAM 4.0 phantom. The threshold image contrast values obtained for the gold disc diameter of 0.10 mm are particularly important in the context of the object thickness and tube voltage compensation test - verifying the correct functioning of the exposure automation system in mammography devices. When analyzing the results obtained with CDMAM Analysis v2.3.0 (NCCPM) software for a gold disc for a diameter of 0.10 mm, no statistically significant difference between the obtained threshold image contrast results can be observed. In addition, the lower value obtained with the CDMAM 4.0 phantom is more favorable from the point of view of the obtained CNR (contrast noise ratio) values in the object thickness and tube voltage compensation test. In the case of the remaining diameters, a statistical difference is found between the obtained results. The maximum difference was reached for a gold disc with a diameter of 1.00 mm and amounted to 11.4%. The average difference between the threshold image contrast obtained with CDMAM 3.4 and CDMAM 4.0 phantoms using CDMAM Analysis v2.3.0 (NCCPM) software is 7.93%, which is an acceptable level for quality control tests in X-ray diagnostics.

The situation is quite different if we analyze the obtained images of the CDMAM 3.4 and CDMAM 4.0 phantoms with the software provided by the manufacturer, Artinis, i.e., CDMAM Analyser 1.2 and CDMAM4 Analyser 2.4.0.1 (Table 3). The obtained differences between the compared phantoms for gold discs with diameters of 0.10 mm, 0.25 mm, 0.50 and 1.00 mm are as follows: 20.7% (p = 0.000), 54.8% (p = 0.000), 79.4% (p = 0.000) and 85.7% (p = 0.000). In all compared cases, there are statistically significant differences between the obtained values. All the image contrast threshold values measured with the CDMAM 4.0 phantom are greater than those measured with the CDMAM 3.4 phantom. The average reading difference between the compared phantoms is 60.15%. This is a very large difference in the reading, which in most of the available mammography systems, especially those based on the CR detector, may result in the lack of acceptance of the test performed with this phantom. It is worth noting that in this case the images of the CDMAM 3.4 phantom were analyzed with the first software available on the market, which can still be used by a very large number of users of the phantom version 3.4.

It is worth noting that the analysis of the CDMAM 4.0 phantom readings with the CDMAM4 Analyzer 2.4.0.1 software and the CDMAM Analysis v2.3.0 (NCCPM) software gives results that do not differ statistically from each other. Their difference for gold discs with diameters of 0.10 mm, 0.25 mm, 0.50 and 1.00 mm is respectively: 3.6% (p = 0.332), 0.4% (p = 0.970), 1.7% (p = 0.640), 1.6% (p = 0.457). Each software uses the latest source file (CDCOM) to analyze the images. The differences result from the accuracy of elaboration of the obtained data.

The analysis of the doses of ionizing radiation falling on the detector during the CDMAM phantom measurements showed that with the increase of the radiation dose, the values obtained for the gold disc with a diameter of 0.10 mm are smaller. This is a correct relationship, as the increase in the radiation dose increases the threshold image contrast. For Planmed mammography machines, there was a clear correlation between the value obtained from the phantoms and the incident radiation dose. Correlation coefficient R^2^ for the CDMAM phantom 3.4. and CDMAM 4.0 were respectively 0.63 and 0.70 for Planmed mammography machines, and 0.27 and 0.50 for Siemens mammography machines. It can be concluded that in the case of Siemens mammography machines, the correlation for the CDMAM 3.4 phantom was weak. It should be noted that the dose of ionizing radiation (air kerma on the surface of the table) corresponding to 6 cm of breast thickness after compression, and thus used for imaging CDMAM phantoms, was on average 3,99 mGy for Planmed mammography machines and 4,54 mGy for Siemens mammography machines. At the same time, the mean readings from the CDMAM 3.4 and CDMAM 4.0 phantoms were for Planmed 1.011 ± 0.053 and 0.987 ± 0.050 and Siemens 1.191 ± 0.056 and 1.144 ± 0.054, respectively. Planmed mammography machines use less ionizing radiation than Siemens mammography machines, and yet the obtained threshold image contrast is better than in Siemens mammography machines. When analyzing the mammographs of both companies, the CDMAM 3.4 and CDMAM 4.0 phantoms showed the same reading tendency i.e., the values of the readings from both phantoms were lower with the Planmed mammography machines.

The results obtained this work are in correlation with the available research in this field. An article by Strudly, C.J. et al. [20] compared the threshold image contrast readings using the CDMAM 3.4 and CDMAM 4.0 phantoms. In this study, the readings on 9 mammography devices were compared using software developed by the NCCPM (based on file CDCOM 1.6 and CDCOM 4.1.0). In this work, the average difference in readings between the phantoms was up to 15% for the diameters of gold discs in the range of 0.10 to 0.50 mm, and for a gold disc with a diameter of 1.00 mm, a difference of up to 56% was recorded. For a gold disc with a diameter of 0.10 mm, an average difference of 5% was measured. A similar comparative analysis of two types of phantoms was made by Floor-Westerdijk, M.J. et al. [12]. In this case, the average values of 6 CDMAM 3.4 phantoms and 6 CDMAM 4.0 phantoms, which were obtained on one Lorad Selena mammography machine, Hologic Inc., USA, were compared. In this work, the difference in the threshold image contrast between the phantoms CDMAM 3.4 and CDMAM 4.0 was up to 10%, while for the gold disc with a diameter of 0.10 mm it was 8.66%. In this case, the latest software from the manufacturer Artinis was used (based on file CDCOM 1.6 and CDCOM 4.1.0).

## Conclusions

Summing up the results of the above work, it should be stated that there are differences between individual CDMAM 4.0 phantoms, however, these differences are smaller than in the case of the phantom version 3.4. It is recommended to record the serial number of the phantom used in the measurements in the performed tests to identify the phantom. The CDMAM 4.0 phantom, through a larger number of gold discs of different diameters, more accurately analyzes the threshold image contrast. CDMAM Analysis v2.3.0 (NCCPM) software, which analyzes images obtained from both versions of the phantoms, is the recommended software for analyzing images from CDMAM phantoms. The results obtained with this software, especially for a disk with a diameter of 0.1 mm, do not show a statistical difference. It is worth noting that the latest version of the software available on the market should be used for the analysis of CDMAM phantom images. Older versions of the software may give radically different results and contribute to making the wrong decision related to the acceptance of the test results. Although the result of reading the image obtained using the CDMAM phantom is affected by the phantom itself, i.e., the precision of its manufacture and the software used to read it, it is currently one of the best tools for analyzing the threshold image contrast in mammography.
